# Therapeutic efficacy of ozonated blood in severe COVID-19 patients: a randomized controlled trial

**DOI:** 10.3389/fmed.2025.1546767

**Published:** 2025-04-24

**Authors:** Shokrollah Salmanzadeh, Behnam Sheibani, Saeid Bitaraf, Roohangize Nashibi, Sasan Moogahi

**Affiliations:** ^1^Infectious and Tropical Diseases Research Center, Health Research Institute, Ahvaz Jundishapur University of Medical Sciences, Ahvaz, Iran; ^2^Infectious and Tropical Diseases Ward, Razi Teaching Hospital, School of Medicine, Ahvaz Jundishapur University of Medical Sciences, Ahvaz, Iran; ^3^Department of Community Medicine, School of Medicine, Ahvaz Jundishapur University of Medical Sciences, Ahvaz, Iran

**Keywords:** COVID-19, SARS-CoV-2, ozone therapy, immunology, reactive oxygen species, length of stay, toxicology, complementary therapies

## Abstract

**Introduction:**

During the COVID-19 pandemic, several studies were published on the use of ozone therapy in treating COVID-19, leveraging pre-pandemic published data on the anti-inflammatory, antimicrobial, and immunomodulatory properties of ozone gas. In this pilot randomized controlled trial conducted during the pandemic, we aimed to assess the outcomes of blood ozone therapy (OT) as an investigational agent versus the COVID-19 standard of care as standalone on 60 patients diagnosed with severe COVID-19.

**Methods:**

This study was conducted as a randomized controlled trial in which both arms of the study received the Iranian Health Ministry’s COVID-19 treatment guideline as the standard of care; the intervention group additionally received intravenous ozonated blood based on the related international society guidelines.

**Results:**

Our findings revealed a statistically non-significant 33% higher hazard ratio for a prolonged hospital stay in the OT group. However, the OT arm exhibited a significantly higher odds ratio of 4.3 for ICU transfer of patients initially admitted to general wards. The univariate logistic regression analysis of mortality found a 3.5-fold increased probability associated with OT use, though this difference was not statistically significant.

**Conclusion:**

We suggest that further trials with robust study designs utilizing larger populations are required to further assess the role of OT on severe COVID-19 keeping in mind a heightened awareness of potential unfavorable outcomes throughout the study.

**Clinical trial registration:**

https://irct.ir, identifier IRCT20200616047792N1.

## 1 Introduction

The COVID-19 pandemic resulted in over 7.1 million deaths worldwide as of 20 February 2024 according to the World Health Organization (1). The infection-hospitalization ratio (IHR) for COVID-19 is reported at an overall 2.1% with an age-related variation. Approximately 25% of hospitalized patients required ICU care and 15% required intermittent mechanical ventilation (2). The leading causes of death in COVID-19 patients are respiratory failure and severe inflammation leading to a cytokine storm with multiple organ failure (3). The onset of the pandemic led to the employment of various therapeutic strategies including the use of pre-existing antivirals, immunomodulators, and alternative treatments such as ozone therapy (4, 5) The interest in the application of ozone therapy in COVID-19 arises from theories and ongoing research on its anti-inflammatory, immunomodulating, and antimicrobial effects (6). Over the last decade, positive evidence has been collected on the use of this treatment method in healing ulcerative wounds, treating osteoarthritis, and pain control in musculoskeletal rehabilitation (7). However, with regards to the use of ozone gas as therapy for COVID-19, this agent remains an investigational product.

Ozone therapy refers to administering a gas mixture of 97% oxygen plus 3% ozone gas to a patient through various possible routes utilizing concentrations of 10–70 μg/ml of ozone gas, the latter being the typical concentrations used for medical purposes (8). Ozone gas is produced by passing pure oxygen through a medical ozone generator with a high voltage gradient (5–13 KV). Medical ozone gas can be applied systemically or locally. The most common form of systemic administration is intravenous ozonated auto-hemotherapy, which involves drawing a patient’s blood sample, admixing it with ozone gas, and reinfusing it into the patient’s bloodstream (9, 10). Overall, ozone therapy has been shown to be safe when administered correctly and within recommended doses in several observational studies, randomized clinical trials, and meta-analyses (11).

Since the onset of the COVID-19 pandemic, several studies have suggested intravenous ozonated auto-hemotherapy as a potential therapeutic tool for mild, moderate, and even severe COVID-19 patients (12–16). To date, the number of studies on the use of ozone therapy on COVID-19 is limited, and questions regarding efficacy and even safety remain. This pilot clinical trial aimed to contribute to the current assessment of the response to intravenous ozone auto-hemotherapy with a specific focus on severe COVID-19 population group. For the purpose of this study and ease of reference, the term “intravenous ozonated auto-hemotherapy” will be referred to as “ozone therapy” (OT) throughout this article.

## 2 Materials and methods

For the reason of a scarcity of available data on the efficacy and safety of ozone therapy on COVID-19 patients, we implemented this study in the form of a pilot parallel randomized-controlled trial (RCT). This study was carried out at Razi (Rhazes) General Hospital in Ahvaz, province of Khuzestan in southwestern Iran. The enrolled patients were randomized in blocks of six and equal numbers into both arms of the study. This RCT was approved by the Ethics Committee of Ahvaz Jundishapur University of Medical Sciences (IR.AJUMS.REC.1399.217) and conducted based on the principles of the Declaration of Helsinki. Written informed consent was obtained from all patients. Finally, this study was listed at the Iranian Registry of Clinical Trials (registration number IRCT20200616047792N1), accessible at https://irct.ir. Following the latter and the initiation of patient recruitment, changes in patient selection criteria and methods were not made. In this study, an interim analysis was not carried out.

Razi Hospital’s infectious diseases ward is the academic and referral center for the province (population of 4.5 million) and has been the COVID-19 referral center for Ahvaz (population of 1.18 million) since the advent of this pandemic (17). We enrolled adults aged 18–75 diagnosed with severe COVID-19 admitted at Razi hospital through the emergency department to either the infectious diseases ward or the intensive care units.

All patients included in this trial were confirmed cases of COVID-19 infection (diagnosed by nasopharyngeal swab and PCR performed on admission) with severe pneumonia with a saturation of oxygen as measured by pulse oximetry (SpO_2_) ≤ 93% on room air at sea level in combination with any of the following: a respiratory rate > 24 breaths/min, blood pressure less than 90/60, decreased level of consciousness or chest CT changes in the form of peripheral unilateral or bilateral basal patches of ground-glass opacity(s). Of note, the criterion of SpO_2_ ≤ 93% on room air at sea level best corresponds with the United States (US) National Institute of Health (NIH) definition of severe COVID-19.

Patients with thrombocytopenia, hemophilia or other coagulopathies, a past medical history of seizure, hypothyroidism, pancreatitis, acute alcohol poisoning, known allergy to ozone gas or ozonated products, and if pregnant or breastfeeding were excluded from the trial as advised in released practice protocols by societies endorsing standardization of practice in ozone therapy, specifically the International Scientific Committee on Ozone Therapy (ISCO3), and the World Federation of Ozone Therapy (WFOT) (18, 19).

The control group received the standard of care (SoC) COVID-19 treatment protocol from the Iranian Ministry of Health (20) (Table 1). The intervention group, in addition to the Iranian SoC treatment protocol, received ozone therapy wherein 200 ml of patient blood was drawn into a heparinized sterile container and admixed with 120 ml of 30 μg/mL of ozone gas for 5 min, which was then transfused back into the patient’s bloodstream over 15 min; this protocol is based on the ISCO3 and WFOT guidelines (18, 19). The device used for ozone generation was Medozon Compact manufactured by Herrman Apparatebau GmbH, Elsenfeld, Germany capable of producing ozone gas with a concentration ranging from 2 to 80 μg/ml. This device does not utilize a spectrophotometer. The planned sessions of OT were three sessions per week with a day of rest between each episode for a maximum total of 6 administered sessions. If a patient reached an improved oxygenation status to the point that would enable his/her discharge in less than 2 weeks, fewer sessions of OT were delivered.

**TABLE 1:** The Iranian Ministry of Health standard of care treatment for severe COVID-19.

Standard of care delivered to patients in both arms of the study
Dexamethasone 8 mg daily IV injections for 10 days
Heparin 5000 IU SC TDS or enoxaparin 60 IU SC daily
Remdesivir 200 mg IV on day 1 then 100 mg for 4 days

The primary outcome assessed in this study was the duration of hospital stay for each patient, defined as the date of admittance up to the time of discharge or decease. The evaluated secondary outcomes include the ICU transfer rate, the case fatality rate, the daily changes in oxygenation index (as blood oxygen saturation and respiratory rate and supplemental oxygen requirement), and every other day measurements of ESR, CRP, lymphocyte count, hemoglobin, platelet count, LDH, D-Dimer, liver function tests, and INR.

**FIGURE 1:**
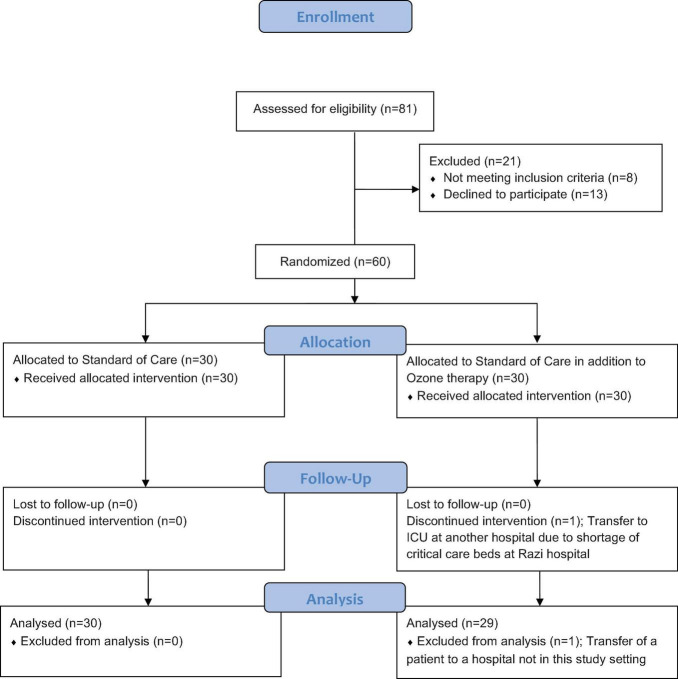
Flow diagram for patient enrollment.

A total of 60 patients who met the study criteria were enrolled in this trial and assigned to the intervention and control groups by block randomization, with 30 participants in each group. Randomization was based on the block-chain method, and the individuals were randomly divided into two groups based on 10 blocks of six (with each block carrying a 1:1 ratio of control and intervention members) by a person who was not involved in the study process. The block randomization schedule was obtained from www.sealedenvelope.com. Due to the nature of the intervention, blinding was not possible. The CONSORT flow of this randomized trial is presented in Figure 1. In this study, one patient on the intervention arm who developed a need for ICU care, with all such beds being full at Razi Hospital, was transferred to another hospital and was hence excluded from the final data assessment.

The demographic and primary data were analyzed using descriptive statistics, including frequency and percentage, average, and standard deviation. Survival analysis, Cox regression, and the Kaplan-Meyer chart were used to analyze the primary outcome: the length of hospital stay. Logistic regression was used to compare mortality between the two groups. The general evaluation equation (GEE) was used to compare measured laboratory parameters between the two groups due to repetitive measurements. All analysis was performed using Stata version 13.1.

## 3 Results

In this study, 81 patients diagnosed with severe COVID-19 were approached. Eight patients had to be precluded, having one or more of the exclusion criteria, and 13 further patients declined to offer consent and were excluded. Four patients, one in the control group and three in the intervention group, were directly admitted to the ICU; none of the four patients were in organ failure or septic shock and did not require ventilation. The patient recruitment period was from 18 Aug 2020 to 20 Sep 2020.

The mean age of participants in the control group (18 males, 12 females) was 58.07 ± 11.30 years, whereas this figure was 54 ± 12.47 years for the OT group (22 males, eight females) (Table 1). There was an uneven distribution between two baseline characteristics, namely gender and hypertension, but both were controlled for as confounding variables. The patients’ mean primary chest CT scores at admission for the control and OT groups were 11.33 ± 3.07 and 10.73 ± 3.08, respectively (21) (Table 2). There was no interchange of patients between the two arms of the study.

**TABLE 2:** Baseline demographic and clinical characteristics of sampled patients in both groups of study.

Parameter	Control group (*n*:30)	Ozone group (*n*:30)
Age (mean ± SD)	58.07 ± 11.30	54 ± 12.47
**Gender**		
Male	18 (60%)	22 (73%)
Female	12 (40%)	8 (27%)
**Co-morbidities**		
Hypertension	12 (40%)	5 (17%)
Diabetes mellitus	9 (30%)	6 (20%)
Obesity	2 (7%)	1 (3%)
Cardiovascular disease	4 (13%)	1 (3%)
Several diseases	8 (27%)	5 (17%)
Primary chest CT score (mean ± SD)	11.33 ± 3.07	10.73 ± 3.08

### 3.1 Primary outcome

**FIGURE 2:**
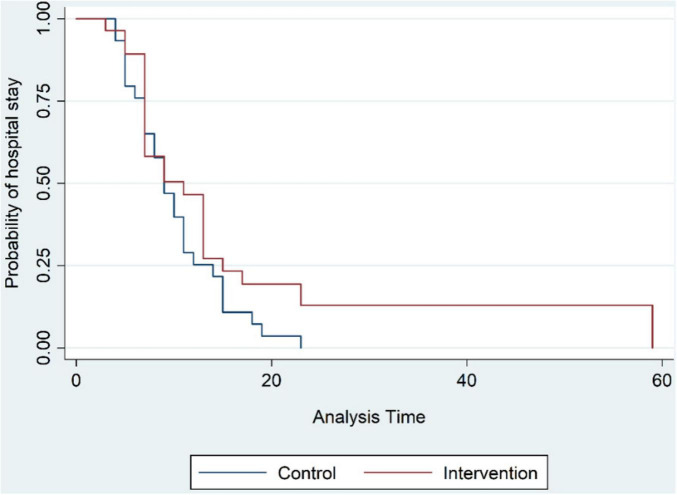
The Kaplan-Meier estimation of the duration of hospitalization.

The mean duration of hospital stay as the primary investigated outcome was 9.7 ± 4.9 days for the control group, while this number was 12.2 ± 10.86 days for the OT group, a mean increase of 2.5 days (Figure 2). Thereby, the hazard ratio for a lengthened hospital stay for patients who received OT was 33% greater than the patients who did not receive this intervention, but this difference was not statistically significant (HR = 0.769, 95% CI = 0.455–1.3, *p*-value = 0.326); this relationship remained non-significant when data were corrected for observed differences in gender and hypertension between the two groups. Similarly, the duration of hospital stay precluding patients admitted directly to the intensive care units was longer for the intervention group through not by a statistically significant difference the data for which is available in Table 2 of the supplementary data section.

### 3.2 Secondary outcomes

On the secondary outcomes, the development of the need for ICU care of patients initially admitted to general ward was evaluated. In analyzing this outcome, four patients who had been directly admitted to the intensive care units from the emergency room were excluded. Of the 56 remaining patients, three patients (10.3%) from the control group and 11 patients (42.3%) from the OT group required transfer to the special care units: the rate of ICU transfer from the ward was significantly higher for the OT group (Odds ratio = 4.3, 95% CI = 1.16–15.85, *p*-value = 0.042).

On the mortality rate, a univariate analysis through logistic regression revealed that the odds of death for the OT group were 3.5 times greater than the control group, though this difference was statistically non-significant (Odds Ratio = 3.5, 95% CI = 0.64–18.98, *p*-value = 0.146). The control group had two deceased patients (ages 46 and 71 years old), whereas the intervention group had six (ages between 52 and 70 years old). The cause of death in all patients across both groups was respiratory or multi-organ failure.

The evaluation of the patient oxygen requirement as the respiratory rate and blood oxygen saturation set forth no difference of significance between the control and OT groups.

Of note, our every-other-day measurements of serum hemoglobin levels, thrombocyte count, prothrombin time, partial thromboplastin time, serum creatinine, and liver enzymes displayed no adverse changes secondary to OT.

Lastly, according to our outcome results, the mean and standard deviation for the intervention group in order are 12.2 and 10.86, and 9.7 and 4.2 in a similar order for the comparison group; the probability of Type I error is 0.05, and the power of the study is 0.23. To achieve a performance of at least 0.8, we would need 176 participants in each group.

## 4 Discussion

The global effort to combat COVID-19 has yielded significant results at two frontiers of prevention and treatment (22). In the context of the latter, complementary and alternative treatments have also garnered consideration. With this pilot study, we aim to assess the effects of ozone therapy (OT) as an alternative treatment, specifically focusing on its impact on severe COVID-19. The interest in this topic stems from numerous published review articles on the use of OT on COVID-19 recommending further randomized controlled trails (RCTs) to draw conclusive evidence (23–25).

The premise for the use of OT in COVID-19 is based on literature outlining two *in vivo* mechanisms of action of ozone gas on SARS-CoV-2, one through oxidative events and the other via modulation of inflammation (26–28). The oxidative mechanisms include direct oxidation of the S-Protein interrupting virus cell entrance and disrupting viral integrity via reaction with polyunsaturated fatty acids and the amino acids methionine, tryptophan, and cysteine (15, 27). OT also interferes with viral replication through the direct oxidation of cysteine residues (26). In addition, OT is noted to activate reactive oxygen species (ROS), which indirectly inactivate the virus (15, 27). Regarding regulation of inflammation, as the second mechanism of action of OT there is the modulation of the NLRP Inflammasome, IFN-γ, IL-2, and TNF-α, and activation of Nrf2 leading to suppressed production of proinflammatory cytokines via inhibition of NF-κB and impaired SARS-CoV-2 replication (16, 26, 28–30).

The primary limitation of this study pilot trial is the small population size. The power analysis for the estimated required sample size indicated a need for 176 participants per group. Therefore, our limited population size confines the possibility of a comprehensive evaluation of the task at hand.

In evaluating our primary outcome, we observed a 33% increased hazard for prolonged hospital stay in the intervention arm; however, this increase was statistically insignificant. In this regard, since in the intervention group, three patients were directly admitted to the intensive care units versus only one patient from the control group, it may be reasoned that the three patients were prone to longer hospital stays hence effecting our primary outcome. To address this, a further analysis withholding the above four patients similarly yielded a non-significant prolonged hospital stay for the intervention group. Amongst the secondary outcomes in this study, a finding which was statistically significant was development of a need for ICU care of patients initially admitted at general ward, three patients (10.3%) in the control group and 11 (42.3%) in the OT group required ICU transfer, resulting in a 4.3 times greater odds ratio of ICU transfer for the OT group.

To date, there are nine original articles evaluating the efficacy of intravenous ozone auto-hemotherapy (OT) on COVID-19 (31–39). Of the nine studies, four were randomized and controlled (RCT) where one of the four RCTs covered only the mild to moderate COVID-19 patients; inclusion of mild COVID-19 cases in the study may be considered a confounding factor in that the mild cases follow a benign clinical course the majority of which require only symptomatic intervention (40). Therefore, of the nine studies there were only three RCTs evaluating the severe form of COVID-19. Of the non-randomized studies, three were only controlled and one was neither randomized nor controlled. A summary of all mentioned studies above is provided in Supplementary Table 1.

The RCTs on severe COVID-19 differ in their approaches; Sozio et al. with a population of 48, on a three consecutive day approach deliver 8 mg of Ozone gas per session while Araimo et al. in a study of 28 patients deliver 7.5 mg of ozone gas per session for 7 days, and Aghamohammadi et al. on a population of 48 critical COVID-19 patients opt for a thrice a week (total of 10 sessions) delivery of 3 mg of ozone gas per session (33, 35, 39). In reference to the primary outcome of our trial, while we report a non-significant increased risk of longer hospital stay, on inspecting the above RCTs, Sozio et al. report no change in hospital stay with the use of OT (35). On mortality rates, where Araimo et al. and Sozio et al. report no significant difference in the fatality rate for the OT group, we report a non-significant increased mortality rate (33, 35). Regarding the need for ICU transfer under OT, Sozio et al. reported no significant difference in the need for ventilatory support between the OT and control arms, whilst we report a significant increased rate of ICU transfer rate (35). Araimo et al. report no effect on mechanical ventilation under OT (33). This is in contrast to the findings in the RCT by Aghamohammadi et al. on critical COVID-19 patients under mechanical ventilation, in which a significantly shorter intensive care unit stay and more ventilation-free days under OT is reported (39). On inflammatory markers, Araimo F et al. similarly to our findings report no significant effect under OT (33). At this point, it appears fair to argue that the evidence originating from RCTs on the use of OT as a treatment modality specifically on the severe disease form of COVID-19, is still assimilating and as of yet incongruent.

Regarding the control-only studies of OT on COVID-19, the results are variant. Izadi et al. and Hernandez et al. report a decrease in inflammatory markers while Çolak et al. reported no change in this criterion. Hernandez et al. reported a shortened hospital stay while Çolak et al. reported no change in ICU transfer rates. And one study by Tascini et al. as a sole finding reported only a decrease in oxygen requirement under OT. The control-only studies of OT on COVID-19 which compromise five of the nine available studies, the results here are also variant. Izadi et al. and Hernandez et al. report a decrease in inflammatory markers while Çolak et al. reported no change in this criterion (31, 34, 38). Hernandez et al. reported a shortened hospital stay while Çolak et al. reported no change in ICU transfer rates (31, 34). And one study by Tascini et al. as a sole finding reported a decrease in oxygen requirement under OT (36).

The severe form of COVID-19 consist of two disease phases: an initial viral phase transiently replaced by an inflammatory stage, where the latter may descend further into a cytokine storm (41, 42). The current postulations on the mechanism of action of ozone therapy predominantly suggest two routes of action, one via an antiviral, pro-oxidative arm and the other through an anti-inflammatory arm (15, 16, 26–28, 43). However, there is a lack of a clarity as to which of the two arms predominates over the other. And considering the distinct inflammatory nature of severe COVID-19, it can be hypothesized that the oxidative properties ozone gas, may potentially exacerbate inflammation in severe COVID-19. Moreover, ozone gas has a hormetic effect and is dose dependent, further impeding interpretation the actual actions of ozone in the body (44). Given the paucity of well-structured studies, limitations in study populations and the variance in results, it is evident that further trials with larger populations under robust study designs are required to further assess the role of OT on severe COVID-19.

## 5 Conclusion

The data on ozone therapy for severe COVID-19 is still emerging. We recommend further trials with robust designs, larger populations, utilizing precautionary measures to further evaluate the effectiveness of ozone therapy on severe COVID-19 and the potential for unfavorable outcomes.

## Data Availability

The raw data supporting the conclusions of this article will be made available by the authors, without undue reservation.
