# Supplementary material for: Therapeutic efficacy of ozonated blood in severe COVID-19 patients: a randomized controlled trial
Source: Front Med (Lausanne). 2025 Apr 24;12:1546767. doi: 10.3389/fmed.2025.1546767 (PMC12058501; doi:10.3389/fmed.2025.1546767)
Supplement: Supplementary file 1 [file Table_1.docx]

**Supplement Table 1.** Studies evaluating the therapeutic effect of ozone auto-hemotherapy on severe COVID-19

| **Authors** | **The severity of COVID-19 Studied (mild, moderate, or severe)** | **Statistically significant response(s) to ozone therapy** | **Randomized** | **Controlled** | **Sample size** | **Daily dosage of ozone gas delivered** | **No. of days of ozone therapy** | |
| --- | --- | --- | --- | --- | --- | --- | --- | --- |
| Hernandez A, et al | Only severe cases | Shorter hospital stay, decreased inflammatory markers, decreased oxygen requirement | No | Yes | 18 | 16mg | | 5 |
| Shah M, et al | Mild to moderate cases | Improved cough and breathlessness, decreased inflammatory markers, reduced transfer to ICU | Yes | Yes | 60 | 12mg | | 10 |
| Araimo F, et al | Only severe | No sig effect on inflammatory markers, No sig effect on the need for ventilatory support, No sig effect on mortality | Yes | Yes | 28 | 7.5mg | | 7 |
| Çolak S, et al | Mild to severe cases | Lower mortality, No sig effect on inflammatory markers, No sig effect on ICU transfer | No | Yes | 53 | 3mg | | Not specified |
| Sozio E, et al | Moderate and severe cases | No sig effect on length of hospital stay, No sig effect on ICU admission, No sig effect on mechanical ventilation requirement, No sig effect on mortality rates, Decrease in oxygen requirement | Yes | Yes | 48 | 8mg | | 3 |
| Tascini C, et al | Moderate to severe | Decrease in oxygen requirement | No | Yes | 60 | 8mg | | 3 |
| Franzini M, et al | Severe to Critical | Decrease in oxygen requirement, decreased inflammatory markers | No | No | 50 | 9mg | | 5 |
| Izadi M, et al | Moderate to severe | Decrease in inflammatory cytokines | No | Yes | 200 | 9mg | | 5-10 |
| Aghamohammadi D, et al | Critical | Shorter ICU stay, more ventilation-free days | Yes | Yes | 40 | 9mg | | 10 |
