# Supplementary material for: Therapeutic efficacy of ozonated blood in severe COVID-19 patients: a randomized controlled trial
Source: Front Med (Lausanne). 2025 Apr 24;12:1546767. doi: 10.3389/fmed.2025.1546767 (PMC12058501; doi:10.3389/fmed.2025.1546767)
Supplement: Supplementary file 2 [file Table_2.docx]

**Supplement** **Table 2.** Duration of hosptial stay; whole study population vs. whole population precluding patients directly admitted to the intensive care

|  | Control size | Intervention size | Median stay: Control group | Median stay: Intervention group | Difference in hospital stay | p value |
| --- | --- | --- | --- | --- | --- | --- |
| Whole Study population | 30 | 29 | 9.7 | 12.207 | -2.507 | 0.255 |
| Study population precluding direct ICU admissions | 29 | 26 | 9.69 | 11.846 | -2.156 | 0.359 |
